# HOSPITALIZED AFTER A FALL: EXAMINING THE IMPACT OF INPATIENT GERIATRIC POLYPHARMACY REVIEW AND DEPRESCRIBING

**DOI:** 10.1093/geroni/igad104.3395

**Published:** 2023-12-21

**Authors:** Pratima Gangupantula, Alissa Cooney

**Affiliations:** University of Texas Health Science Center San Antonio, San Antonio, Texas, United States; University of Texas Health Science Center San Antonio, San Antonio, Texas, United States

## Abstract

Falls are reported in 1 out of 3 older adults (≥65 years old) annually. While there are many modifiable factors contributing to falls in OA, polypharmacy increases risk of falling as well as recurrent falls by 1.5-2x. In an acute inpatient setting, geriatricians can evaluate an OA’s medications to ensure these medications are indicated and potential benefits outweigh potential risks. Little is known regarding the impacts of deprescribing (including discontinuing, holding, or tapering medications) in the inpatient setting for OA admitted for falls. A retrospective chart review was conducted at a single academic institution from January 1st to June 30th 2023 to examine associated polypharmacy and deprescribing in OA hospitalized after a fall. The OA’s complete home medication list was obtained, geriatric consultation notes were reviewed to determine if deprescribing was recommended, and commonly deprescribed drugs were noted. Of the 283 OA meeting inclusion criteria, 238 (84.45%) OA experienced polypharmacy. Deprescribing recommendations were given to 203 (84.94%) of OA experiencing polypharmacy. 54.39% of OA had ≥1 medications discontinued, 71.13% of OA had ≥1 medications held, and 23.85% of OA had ≥1 medications tapered. Commonly deprescribed drug classes included antihypertensives, non-steroidal anti-inflammatory drugs, proton pump inhibitors, and opioids. Pantoprazole, aspirin, omeprazole, and zolpidem were the most commonly deprescribed Beers criteria medications. Polypharmacy is associated with deprescribing in older adults admitted for falls. Future studies should examine whether deprescribing recommendations were followed by healthcare providers when given in an inpatient setting and whether deprescribing contributes to decreased fall risk over time.

